# Case Report: Combined pembrolizumab, 5-fluorouracil, and cisplatin therapy were remarkably effective in p16-positive squamous cell carcinoma of unknown primary

**DOI:** 10.3389/fonc.2023.1231986

**Published:** 2023-07-10

**Authors:** Risako Suzuki, Kazuyuki Hamada, Ryotaro Ohkuma, Mayumi Homma, Toshiaki Tsurui, Nana Iriguchi, Tomoyuki Ishiguro, Yuya Hirasawa, Hirotsugu Ariizumi, Yutaro Kubota, Atsushi Horiike, Kiyoshi Yoshimura, Satoshi Wada, Toshiko Yamochi, Takuya Tsunoda

**Affiliations:** ^1^ Division of Medical Oncology, Department of Medicine, Showa University School of Medicine, Tokyo, Japan; ^2^ Department of Chest Surgery, Fukushima Medical University, Fukushima, Japan; ^3^ Department of Pathology, Showa University School of Medicine, Tokyo, Japan; ^4^ Department of Clinical Immuno Oncology, Clinical Research Institute for Clinical Pharmacology and Therapeutics, Showa University, Tokyo, Japan; ^5^ Department of Clinical Diagnostic Oncology, Clinical Research Institute of Clinical Pharmacology and Therapeutics, Showa University, Tokyo, Japan

**Keywords:** cancer of unknown primary, p16, 5-fluorouracil, cisplatin, PD-1 antibody, pembrolizumab, squamous cell carcinoma

## Abstract

**Background:**

Cancer of unknown primary (CUP) is a malignant tumor without a known primary lesion with a frequency of 3−5%. It can be divided into favorable and unfavorable prognosis subsets. While recommended treatments are available for the former group, there is no established treatment for the latter. Here, we report the effective treatment of a 32-year-old woman with p16-positive squamous cell CUP with pembrolizumab plus 5-fluorouracil and cisplatin therapy.

**Case presentation:**

A 32-year-old woman presented with metastatic lesions in the liver, lung, bone, cervical region, abdominal region, and pelvic lymph nodes. She was diagnosed with p16-positive squamous cell carcinoma of unknown primary origin. The patient received pembrolizumab plus 5-fluorouracil and cisplatin therapy, which markedly reduced the metastasis and improved her Eastern Cooperative Oncology Group performance status after two courses.

**Conclusion:**

This case report highlights the potential of pembrolizumab plus 5-fluorouracil and cisplatin therapy for treating CUP with an unfavorable prognosis. p16 positivity is worth examining for squamous cell carcinoma of unknown primary origin, and if present, this therapy should be considered a promising treatment option.

## 1 Introduction

Cancer of unknown primary (CUP) is a malignant tumor histologically defined as a metastatic lesion whose primary lesion is unknown, despite sufficient investigations (1). The frequency of CUP is 3−5% of all malignant epithelial tumors (2), the median survival time is 6−9 months, the 5-year survival rate is 2–6%, and the 1-year survival rate from diagnosis is approximately 50% (2, 3).

The concept of an ‘unknown primary’ also exists in malignant melanomas, with approximately 3% of melanomas classified as melanoma of unknown primary (MUP) lacking an identifiable primary site. A recent publication suggests that MUP may have a better prognosis compared to melanoma of the same stage but with a known primary site. This is likely attributable to its high immunogenicity, which is reflected in the immunologically mediated regression of the primary site (4).

Patients with CUP are categorized into two prognostic subsets: favorable prognostic subsets (20%) and unfavorable prognostic subsets (80%) (5). The prognosis is generally poor; however, some groups are curable and have a good prognosis.

Diseases in the favorable prognosis subset include adenocarcinoma with only axillary lymph node metastasis in women, adenocarcinoma with only peritoneal metastasis and increased CA125 in women, adenocarcinoma with only bone metastasis and increased prostate-specific antigen in men, and squamous adenocarcinoma with only lymph node metastasis, such as cervical and inguinal lymph nodes (6). Furthermore, a recent report suggests that the CUP subset thought to share similar properties with colorectal, lung, and renal cancer based on immunostaining results represents a new subset associated with a favorable prognosis (7). While there are recommended treatments for patients in the favorable prognostic subset, treatments for the unfavorable prognosis subset have not been established. Data regarding previously published case reports are shown in **Table 1** (8–38). Most cases in the table are adenocarcinomas or poorly differentiated cancers. Combination therapy with platinum has mainly been used in such cases. There are no reports on pembrolizumab plus 5-fluorouracil and cisplatin therapy for p16-positive squamous cell carcinoma of unknown primary origin.

**Table 1:** Results of the first-line treatment regimens reported for groups with unfavorable prognoses for carcinoma of unknown primary (CUP) from 2000 to 2021.

CBDCA-based	Drug	N	RR(%)	mPFS(m)	MST(m)	Reference
Briasoulis (2000)	CBDCA + PTX	77	38.7	NA	13	(8)
Greco (2000)	CBDCA+DTX	47	22		8	(9)
Dowell (2001)	CBDCA+ETP	17	18.8	NA	6.5	(10)
Piga (2004)	CBDCA+DTX+ETP	102	26.5	4	9	(11)
EI-Rayes (2005)	CBDCA+PTX	73	23	NA	6.5	(12)
Pittman (2005)	CBDCA+GEM	51	30.5	4.2	7.8	(13)
Schneider (2007)	CBDCA+GEM+Cape	33	39.4	6.2	7.6	(14)
Pentheroudakis (2008)	CBDCA+DTX	23	17.4	3.1	5.3	(15)
Yonemori (2009)	CBDCA+CPT-11	45	41.9	4.8	12.2	(16)
Huebner (2009)	CBDCA+PTX	46	23.8	6.1	11	(17)
Hainsworth (2009)	CBDCA+PTX+Bev+Er	49	53	8	12.6	(18)
Hainsworth (2010)	CBDCA+PTX+ETP	93	18	3.3	7.4	(19)
CDDP-based						
Voog (2000)	CDDP+ETP	22	32		8	(20)
Greco (2000)	CDDP+DTX	26	26		8	(9)
Parnis et al. (2000)	CDDP+5-FU+EPI	43	23	NA	5.8	(21)
Saghatchian (2001)	CDDP+ETP→CDDP+ETP+BLM+IFM	30	40		9.4	(22)
Saghatchian (2001)	CDDP+5-FU+αIFN	18	44		16.1	(22)
Guardiola (2001)	CDDP+DTX+ETP	22	50	8.8	10.7	(23)
Macdonald (2002)	CDDP+5-FU+MMC	31	27	3.4	7.7	(24)
Culine (2002)	DXR+CPA/CDDP+ETP	82	39	NA	10	(25)
Culine (2003)	CDDP+GEM	39	55	NA	8	(26)
Culine (2003)	CDDP+CPT-11	40	38	NA	6	(26)
Balana (2003)	CDDP+GEM+ETP	31	36.6		7.2	(27)
Park (2004)	CDDP+PTX	37	42	4	11	(28)
Palmeri (2006)	CDDP+GEM+PTX	33	48.5	7	9.6	(29)
Palmeri (2006)	CDDP+PTX+VNR	33	42.3	7	13.6	(29)
Mukai (2010)	CDDP+DTX	45	65.1	5	11.8	(30)
Groaa-Goupil (2012)	CDDP+GEM	27	19	5	11	(31)
Tsuya (2013)	CDDP+S-1	46	41.3	7.5	17.4	(32)
Demirci (2014)	CDDP+DTX	29	37.9	6	16	(33)
L-OHP-based						
Briasoulis (2008)	L-OHP+CPT-11	47	13	2.7	9.5	(34)
Shin (2016)	L-OHP+5-FU(FOLFOX6)	23	35	3	9.5	(35)
Other						
Dowell (2001)	PTX+5-FU	17	18.8	NA	8.4	(10)
Hainsworth (2007)	Bev+Er	51	10	3.9	7.4	(36)
Huebner (2009)	GEM+VNR	46	20	3.2	7	(17)
Hainsworth (2010)	GEM+CPT-11	105	18	5.3	8.5	(19)
Holtan (2012)	GEM+CPT-11	31	12	NA	7.2	(37)
Tanizaki (2021)	Nivolumab	45	22.2	4	15.9	(38)

RR, response rate; mPFS, median progression-free survival; MST, median survival time; CBDCA, carboplatin; L-OHP, oxaliplatin; PTX, paclitaxel; ETP, etoposide; GEM, gemcitabine; CPT, irinotecan; DTX, docetaxel; Cape, capecitabine; Bev, bevacizumab; Er, erlotinib; BLM, bleomycin; IFM, ifosfamide; αIFN, αinterferon; MMC, mitomycin; VNR, vinorelbine; NA, not available. Various treatments have been reported, such as combined platinum-based doublet, non-platinum doublet and triplet, or more.

Although various treatments have been investigated for the unfavorable prognosis group, the currently available studies comparing these site-specific treatments with empirical chemotherapy are severely flawed, as shown in the table. These include issues with patient incidence (oversampling and long-term recruitment of resistant tumor types), study design limitations (observational and questionable trials), heterogeneity among the CUP classifiers (epigene profiling and transcriptome profiling), and non-comparable treatments. A recent review of the CUP literature suggested two comprehensive clinical trial designs: a visionary approach and a pragmatic approach. Both introduced state-of-the-art diagnostic and therapeutic advances to improve the quality of CUP research and the prognosis of patients (39).

p16, also known as cyclin-dependent kinase inhibitor 2A, was discovered in 1994 as a tumor suppressor gene similar to p53 and has attracted attention as a surrogate marker for human papillomavirus (HPV) infection in HPV-related oropharyngeal cancer (40). It has also been reported that squamous cell carcinoma, cervical dysplasia in the precancerous state, and cervical adenocarcinoma are p16-positive (41). Overexpression of the p16 protein is caused by the inactivation of p53 or Rb by HPV infection and may be an indirect indicator of HPV infection (41). It has also been reported that p16 positivity is a good prognostic factor in oropharyngeal cancer (42). Here, we report the effective treatment of a woman with p16-positive squamous cell CUP with pembrolizumab plus 5-fluorouracil and cisplatin therapy.

## 2 Case presentation

A 32-year-old woman presented with a 1-month history of back pain. Contrast-enhanced computed tomography (CT) revealed multiple liver masses; lung nodules; cervical, abdomen, and pelvic lymphadenopathy; and osteolytic changes. The levels of some tumor markers, including NSE, SCC, SPan-1, CA125, human chorionic gonadotropin (hCG), and IL2-receptor, were elevated. In this case, hCG was thought to have been produced by the tumor because the pregnancy test result was negative. A liver biopsy was performed for the histological diagnosis of cancer, revealing squamous cell carcinoma. The immunohistochemistry (IHC) results were AE1/3 (+), CK7 (focal +), CK20 (focal +), P40 (-), p16 (focal +), ER (-), PgR (-), CK5/6 (focal +), HSA (-), P63 (Very few +), GATA3 (-), PAX8 (-), TTF-1 (-), GCDFP15 (-), mammaglobin (-), uroplakin III (-), SALL4 (-), and hCG-β (-). Although p40 and p63 positive images were scarce, CK5/6 was partially positive, and there was a tendency for keratinization. Thus, a diagnosis of squamous cell carcinoma was made, and it was suggested that this was an HPV-associated tumor.

The endometrial cytopathology grading was class II. Moreover, no abnormal findings were observed in the cervix, and the cytopathology revealed negative results. Endometrial biopsy, upper and lower gastrointestinal endoscopy, and mammography revealed no malignant findings. No malignancy was observed following otolaryngology examination.

The p16-positive status indicated HPV-associated squamous cell carcinoma. The results suggested that the candidate primary sites were the head and neck, uterine cervix, anus, vagina, and penis.

Anti-PD-1 antibody monotherapy is effective for cancers of unknown origin. However, there was rapid progression in this case. Thus, we treated the patient with 5-fluorouracil + cisplatin (CDDP), commonly used for squamous cell carcinoma, combined with pembrolizumab, an anti-PD-1 antibody. The pembrolizumab plus 5-fluorouracil and cisplatin therapy has been established as a safe standard therapy for head and neck cancer.

**Figure 1:**
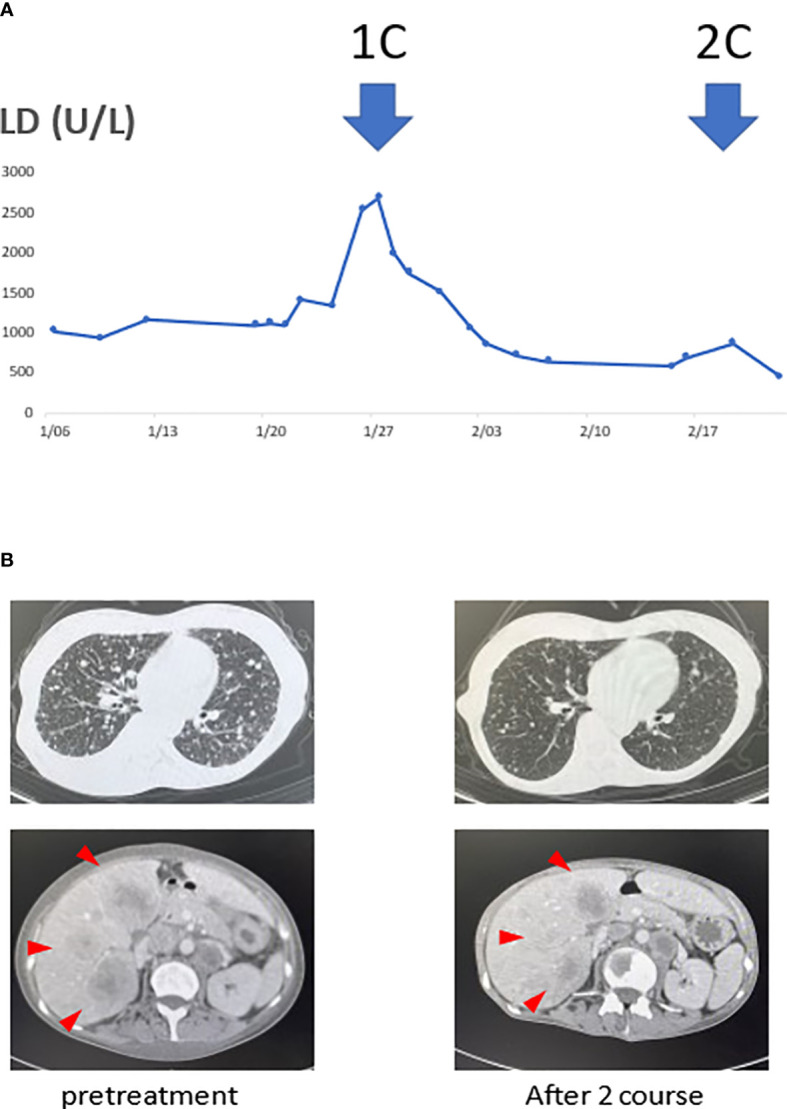
Serum lactate dehydrogenase (LD) levels and tumor volume reduction associated with the treatment. **(A)** The transition of serum LD levels was thought to reflect the disease state. Moreover, computed tomography (CT) scans before treatment and after the two courses showed the tumor. **(B)** The serum LD levels decreased with initiation of treatment. With decreased LD levels, multiple liver and lung metastases also significantly reduced after the two courses.

She received pembrolizumab (200 mg/body intravenously every 3 weeks on day 1), CDDP (100 mg/m^2^ intravenously every 3 weeks on day 1), and 5-fluorouracil (1000 mg/m^2^ as a continuous infusion from days 1−4 every 3 weeks). Her serum lactate dehydrogenase (LD) levels were used to monitor the disease state, as shown in **Figure 1**. After commencing treatment, her LD levels decreased rapidly, and her performance status improved from 2 to 1.

**Figure 2:**
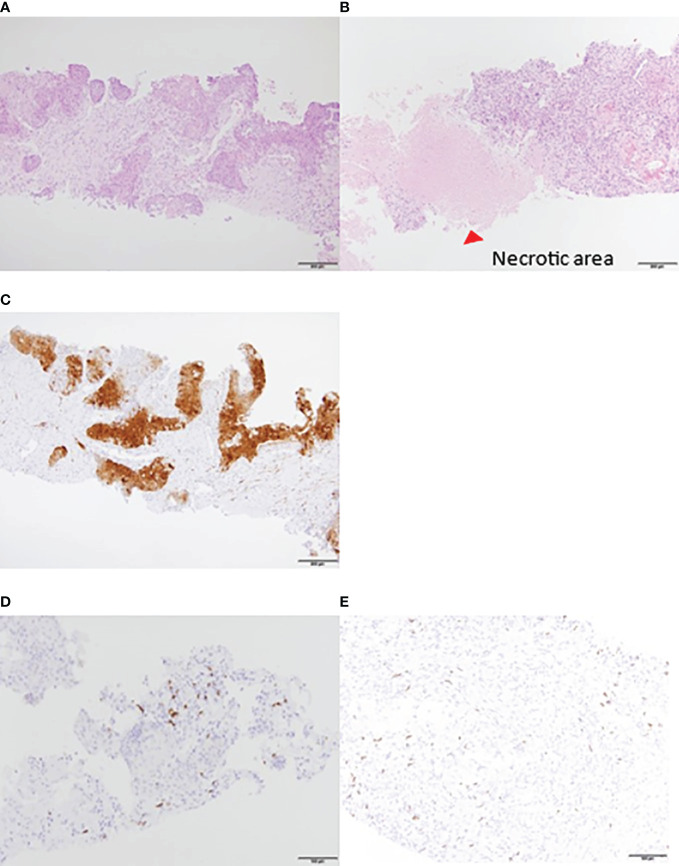
Liver biopsy tissue. HE staining: increased necrotic area after treatment. **(A)** Pretreatment. **(B)** After two courses. IHC: P16 was positive. Local lymphocyte infiltration was observed before the initiation of treatment. **(C)** P16 was positive **(D)** CD8^+^ T lymphocytes pretreatment. **(E)** CD8^+^ T lymphocytes after two courses. Lymphocyte accumulation was observed around the liver. HE, hematoxylin and eosin; IHC, immunohistochemistry.

When a contrast-enhanced CT examination was performed after two courses, the multiple lung, liver, cervical, abdominal, and pelvic lymph node metastases were markedly reduced. She underwent a second liver biopsy after two courses to collect samples for gene panel testing. A liver biopsy before treatment showed CD8+ T cell infiltration in the tumor (**Figure 2D**), and p16 was positive (**Figure 2C**). A second liver biopsy showed increased tumor necrosis (**Figures 2A, B**), while the number of tumor-infiltrating CD8 T cells remained the same as in the previous biopsy (**Figure 2E**).

## 3 Discussion

In this case, we showed that pembrolizumab combined with 5-fluorouracil and cisplatin therapy was effective for p16-positive squamous cell carcinoma, as the treatment rapidly reduced the tumor volume.

There is a need to establish a treatment for CUP. Furthermore, in the era of targeted therapies, the precise histopathological and molecular classification of tumors is crucial to devise the most effective tailored therapeutic strategy. Classifications based on epigenetic alterations have served this purpose. Indeed, cancer cells are characterized by a substantial overall loss of DNA methylation (20–60% overall decrease in 5-methylcytosine) and the simultaneous acquisition of specific hypermethylation patterns at CpG islands of certain promoters. These changes can reversibly or irreversibly alter gene function, contributing to cancer progression (43).

The phase II randomized CUPSICO study (NCT03498521), which is currently ongoing, is comparing the efficacy and safety of targeted therapy or cancer immunotherapy guided by comprehensive genomic profiling versus platinum-based chemotherapy in patients with unfavorable prognosis CUP who have received three cycles of platinum-based induction therapy. The results of this trial may provide a new treatment strategy for CUP (44).

A phase 2 clinical trial investigating the efficacy of nivolumab for CUPs was conducted in 2019 (NivoCUP-2 trial) (45). Fifty-six patients with CUP were included in the study. Forty-five patients were treated, and 11 were untreated. Improvement in the progression-free survival rate was observed regardless of the treatment history. Moreover, the overall survival (OS) rate improved in the treated group but was not reached in the untreated group. The findings from the NivoCUP-2 trial led to the approval of nivolumab as a first-line treatment. However, the response rate was only 18.2%, and the effect of this treatment was inadequate for tumor shrinkage.

Better therapeutic effects were observed in tumors with high PD-L1 expression levels, TMB, and MSI-H. A previous study demonstrated that 28% of patients with CUP showed one or more predictive biomarkers for immune checkpoint inhibitors. In particular, 22.5% of patients had a PD-L1 expression equal to or greater than 5%, 1.8% had MSI-H, and 11.8% had a TMB equal to or greater than 17 per megabase. Patients with CUP and a TMB equal to or greater than ten mutations per megabase tend to have favorable outcomes when treated with immune checkpoint inhibitors (46).

One-third of the cases in the NivoCUP-2 trial had a PD-L1 TPS ≥ 1% (38). A recent case report also showed that when the TMB was high, the combination of pembrolizumab and chemotherapy was effective, even in patients in ‘unfavorable prognosis’ CUP groups (47). It has been reported that viral antigens in anti-PD-1 treatment results are one of the predictive factors (48, 49). In this case, p16 positivity was considered one of the factors that led to a good response.

In general, immune checkpoint inhibitor (ICI) monotherapy requires more time than cytotoxic chemotherapy to achieve therapeutic effects. This is one of the reasons why many studies on ICI monotherapy efficacy show a crossover of survival curves. Considering these ICI characteristics, in this case, the decrease in serum LD levels confirmed immediately after commencing the course and the marked tumor shrinkage in the CT examination at the second course may have resulted from the effects of the cytotoxic therapy.

On the other hand, the liver biopsy results showed lymphocyte accumulation around the liver metastases before the introduction of treatment. Therefore, immunotherapy may have been effective because of the presence of lymphocytes around the lesion.

Recently, the association between the presence of tumor-infiltrating lymphocytes (TILs) and the PD-1 antibody response has been demonstrated. Previous reports have shown that higher levels of TILs provide better therapeutic effects for the PD-1 antibody (50, 51). In this case, the density of the TILs before treatment was high and was maintained at a high level even after commencing treatment. The infiltration of CD8+ T cells into the metastatic liver lesions may have contributed to the effectiveness of the treatment.

The Head and Neck Cancer Guidelines recommend that if p16 is positive, cervical lymph node metastases should be identified and treated as HPV-related oropharyngeal cancer, even if the apparent primary lesion is unknown. The KEYNOTE-048 study, an international phase III trial, was conducted in patients with squamous cell carcinoma of the head and neck to examine the therapeutic effects of pembrolizumab monotherapy and the combined effect of pembrolizumab and 5-fluorouracil + CDDP/CBDCA therapy (52). The study showed a significant prolongation of the OS rate in the chemotherapy and immunotherapy combination group.

In the present case, the patient presented with abdominal pelvic lymphadenopathy, cervical lymph node involvement, and multiple metastases in the liver, lungs, and bones. Although this does not necessarily suggest the high possibility of HPV-related oropharyngeal cancer in this case, it is worth noting that similar treatments have been effective. Pembrolizumab plus 5-fluorouracil and cisplatin therapy may be effective for p16-positive squamous cell carcinoma regardless of an HPV infection.

The limitation is that it is a single case report. We need to further check whether there is a response in other similar cases.

In summary, we recommend that p16 is worth investigating in CUP regarding squamous cell carcinoma. Furthermore, if pathological findings are p16-positive, pembrolizumab plus 5-fluorouracil and cisplatin therapy should be considered a first-line treatment option.

## Data availability statement

The original contributions presented in the study are included in the article/supplementary material. Further inquiries can be directed to the corresponding author.

## Ethics statement

Written informed consent was obtained from the participant/patient(s) for the publication of this case report.

## Author contributions

RS, KH, and RO analyzed and interpreted the patient data and were major contributors to writing the article. MH and TY provided pathological images and contributed to the diagnosis through them. ToT, NI, YH, HA, YK, and AH contributed to data acquisition and analysis. KH, KY, SW, and TaT contributed to the conceptualization and revised the manuscript. All the authors have read and approved the final manuscript.
